# Recurrent Villous Adenoma with High-Grade Dysplasia Arising in a Urethral Diverticulum

**DOI:** 10.1155/2009/361212

**Published:** 2009-06-02

**Authors:** Alireza Zarineh, Elena Bulakhtina, Peter R. Olson, Jan F. Silverman

**Affiliations:** Department of Pathology and Laboratory Medicine, Allegheny General Hospital, Drexel University College of Medicine, 320 East North Avenue, Pittsburgh, PA 15212, USA

## Abstract

Villous adenomas of the urinary tract are an uncommon, well-recognized entity, described in different locations. However, the occurrence of this lesion in the female urethral diverticulum is very unusual. We present the first case of a recurrent villous adenoma with high-grade dysplasia unassociated with adenocarcinoma, arising from a urethral diverticulum. A 75-year-old African-American female presented with urethral prolapse complaining of mild voiding difficulty, stress incontinence, and mild spotting of blood. Histological examination revealed a papillary lesion with finger-like processes lined by pseudostratified columnar epithelium with abundant goblet cells. There were focal areas with stratification to the luminal surface and loss of nuclear polarity and atypical mitoses, interpreted as villous adenoma with high-grade dysplasia. The lesion recurred at one year without evidence of malignant transformation. We also present a brief literature review of urothelial villous adenomas.

## 1. Introduction

Villous adenomas of the urinary tract are a well-recognized but uncommon entity. Several case reports and a number of studies detailing the pathologic features and prognosis of villous adenomas in the urethra, bladder, and urachus have been published [1–4]. However, the histogenesis and malignant potential of these tumors remain controversial. We report a villous adenoma with high-grade dysplasia arising in a 75-year-old African-American female who presented with voiding complaints and mild spotting of blood. The lesion arose in a urethral diverticulum which is exceedingly rare. The villous adenoma also showed high-grade dysplasia which is another uncommon finding. The lesion recurred a year after excision without evidence of malignant transformation. To the best of our knowledge, this is the first reported case in English literature of a recurrent villous adenoma arising in a urethral diverticulum without evidence of malignant transformation. In addition, we reviewed literature, detailing previous studies discussing villous adenomas of the urinary tract.

## 2. Case Presentation

**Clinical Presentation**

A 75-year-old African-American female presented with a urethral mass and evidence of urethral prolapse. She had mild voiding complaints including stress incontinence and mild spotting of blood. Physical examination revealed a mass arising in a diverticulum protruding from the urethra. Cystoscopic examination was performed, and a biopsy was obtained. Subsequently, diverticulectomy was performed. The patient tolerated the operation well and was discharged with no untoward events. The lesion recurred a year after and reexcision was performed. Subsequent 6-month follow-up has shown no evidence of residual neoplasm.

**Pathologic Features**

Gross examination of the biopsy specimens consisted fragments of tan-red tissue, measuring 1.8 × 1.5 cm for excision and 3 × 2 cm for the reexcision specimen.

**Figure 1:**
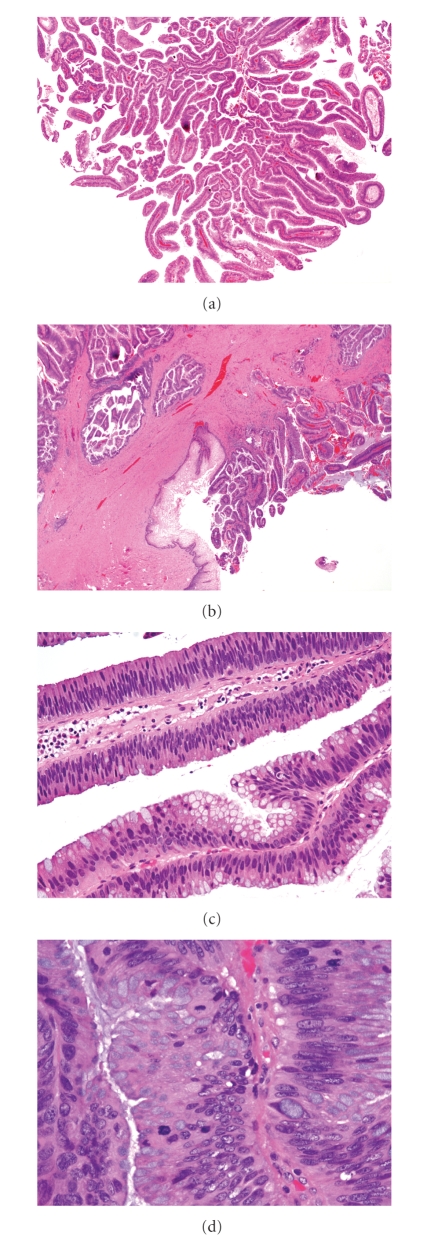
(a) Papillary fronds with delicate fibrovascular core (H&E, X20); (b) border of villous adenoma with squamous epithelium in the urethral diverticulum (H&E, X40); (c) columnar epithelium with abundant goblet cells demonstrating nuclear stratification, surface mitotic figures and loss of polarity (H&E, X100); (d) abundant mitosis, including atypical forms (H&E, X400).

Histologic examination of all specimens revealed a papillary lesion with finger-like processes lined by pseudostratified columnar epithelium with abundant goblet cells. The cells demonstrated nuclear stratification, crowding, hyperchromasia, prominent nucleoli, and abundant mitoses, including atypical forms, interpreted as villous adenoma with high-grade dysplasia (Figures 1(a)–1(d)). Immunohistochemical studies showed diffuse positivity for CK7, CK20, and carcinoembryonic antigen (CEA). The ki-67 index was 20% overall with reaching up to 60% in areas of high-grade dysplasia.

## 3. Discussion

Prior to Cheng et al. report of 23 cases of urinary tract villous adenoma from two large tertiary institutions [5], only isolated cases had been reported, reflecting the rarity of these lesions. The majority of the previous reports note that villous adenomas occur predominantly in the male prostatic urethra, reflecting the rare involvement of the female urethra [3, 6–8]. Thomas et al. studied neoplastic alteration in 90 female patients with urethral diverticulum and found only one villous adenoma among them [9]. Our case is probably the second reported case in English literature. 

Villous adenomas of the urinary tract and the colon share the same microscopic pathologic features. Histologically, villous adenomas from both sites are characterized by papillary projections of pseudostratified columnar epithelium with mucin-producing goblet cells. Cytologic features include nuclear stratification, loss of cellular polarity, and nuclear hyperchromasia. Coexisting invasive adenocarcinoma can be present [4, 5]. Papillary adenocarcinoma can have similar features and may be difficult to distinguish from villous adenoma. 

Immunohistochemical features of adenoma and adenocarcinoma are also similar. Urinary tract adenomas exhibit positive staining for cytokeratin 20 (CK 20) [4, 5] and carcinoembryonic antigen (CEA) [4, 5, 10] but can be epithelial membrane antigen (EMA) negative [4]. Staining for CK 7 and Ki-67 is focally positive [10]. Tumor cells also show positive staining by periodic acid-Schiff (PAS) and Alcian blue at pH 2.5 and pH 1.0 [1]. Expression of epitope for mAbDaS1, usually found on colonic epithelium, was observed in 80% of cases of urethral adenoma in one study [4]. Adegboyega and Adesokan reported DNA aneuploidy and increased expression of p53 in one case of bladder villous adenoma [11].

The exact histogenesis of glandular lesions in the urinary tract is uncertain; however, embryological development of the urinary tract may give some insight into its origin. The cloaca is divided by the urorectal septum into a dorsal rectum and a ventral urogenital sinus. The urogenital sinus gives rise to the majority of the urinary bladder, including the bladder trigone and the neck of the bladder. Remnants of cloacal epithelium may explain the origination of villous adenomas in these structures as well as in the urethra [1, 12]. Another possible mechanism is neoplastic transformation of glandular metaplasia of traumatized urothelium, resulting in villous adenoma [4, 13]. 

The differential diagnoses of villous adenoma of urinary tract include cystitis glandularis and adenocarcinoma. Cystitis glandularis has been reported as a potential mimic of bladder adenocarcinoma [14, 15]. Histologically, it is composed of glands in the lamina propria, which are lined by cuboidal to columnar cells surrounded by one or more layers of urothelial cells. The less frequent intestinal type exhibits colonic type epithelium with goblet cells; however, cystitis glandularis can be easily differentiated from villous adenoma by the absence of villous architecture in the former entity.

The association between villous adenoma and coexistent adenocarcinoma has been established in a number of cases [4, 5]. Though no prospective study has shown if villous adenoma is a precursor of adenocarcinoma, the close association of these two in Cheng et al. series may suggest such a phenomenon [5]. Simple intestinal metaplasia does not seem to be a strong risk factor for bladder adenocarcinoma [16]. 

Prognosis is usually excellent in patients with conventional villous adenoma and those cases demonstrating in situ adenocarcinoma [5]. However, recurrence has been reported in this group of villous adenomas, as opposed to cases not associated with adenocarcinoma in which there is no report of recurrence [13]. Patients are cured by simple excision. However, villous adenoma associated with infiltrating adenocarcinoma requires more extensive treatment. Our case is the first case to demonstrate recurrence without an associated adenocarcinoma, which provides more rationale for previous recommendation of an extended follow-up in these patients postoperatively [9].

In conclusion, we described the first case of recurrent villous adenoma arising in a urethral diverticulum unassociated with adenocarcinoma. It is important to recognize this uncommon lesion which has the potential of malignant transformation and/or recurrence and confusion with the far more common cystitis glandularis.
